# Novel immune-related gene signature for risk stratification and prognosis prediction in ovarian cancer

**DOI:** 10.1186/s13048-023-01289-w

**Published:** 2023-10-19

**Authors:** Hongjun Fei, Xu Han, Yanlin Wang, Shuyuan Li

**Affiliations:** Department of Reproductive Genetics, International Peace Maternity and Child Health Hospital, Shanghai Key Laboratory of Embryo Original Diseases, Shanghai Municipal Key Clinical Specialty, Shanghai Jiao Tong University School of Medicine, No.910, Hengshan Road, Shanghai, 200030 China

**Keywords:** Ovarian cancer, Prognostic risk model, Immune-related genes, Prognosis prediction, Risk score

## Abstract

**Background:**

The immune system played a multifaceted role in ovarian cancer (OC) and was a significant mediator of ovarian carcinogenesis. Various immune cells and immune gene products played an integrated role in ovarian cancer (OC) progression, proved the significance of the immune microenvironment in prognosis. Therefore, we aimed to establish and validate an immune gene prognostic signature for OC patients’ prognosis prediction.

**Methods:**

Differently expressed Immune-related genes (DEIRGs) were identified in 428 OC and 77 normal ovary tissue specimens from 9 independent GEO datasets. The Cancer Genome Atlas (TCGA) cohort was used as a training cohort, Univariate Cox analysis was used to identify prognostic DEIRGs in TCGA cohort. Then, an immune gene-based risk model for prognosis prediction was constructed using the LASSO regression analysis, and validated the accuracy and stability of the model in 374 and 93 OC patients in TCGA training cohort and International Cancer Genome Consortium (ICGC) validation cohort respectively. Finally, the correlation among risk score model, clinicopathological parameters, and immune cell infiltration were analyzed.

**Results:**

Five DEIRGs were identified to establish the immune gene signature and divided OC patients into the low- and high-risk groups. In TCGA and ICGC datasets, patients in the low-risk group showed a substantially higher survival rate than high-risk group. Receiver operating characteristic (ROC) curves, t-distributed stochastic neighbor embedding (t-SNE) analysis and principal component analysis (PCA) showed the good performance of the risk model. Clinicopathological correlation analysis proved the risk score model could serve as an independent prognostic factor in 2 independent datasets.

**Conclusions:**

The prognostic model based on immune-related genes can function as a superior prognostic indicator for OC patients, which could provide evidence for individualized treatment and clinical decision making.

**Supplementary Information:**

The online version contains supplementary material available at 10.1186/s13048-023-01289-w.

## Introduction

Ovarian cancer (OC) is the fifth leading cause of cancer death in women which lead to 5% women die of it in 2021. According to statics from American, there are 21,410 new cases and 13,770 deaths in 2021 [1]. The incidence of OC is considerably lower than the first most common female malignancy breast cancer, but the mortality is three times than breast cancer, even worse, the mortality rate of OC is predicted to be rise significantly in 2040 [2]. The reason of high mortality rate of OC due mainly to lack of effective screening means and prognosis evaluating tools that result in its diagnosis in the advanced stages and harder to treat [3].

Over the past decade, precision diagnostics and treatment strategies in ovarian cancer offer opportunity to improve survival [4]. Meanwhile, advances in precision oncology strategies have increased a need to identify clinically relevant predictive biomarkers within tumours and the best possible candidates for therapies have become more important [5]. Precision cancer therapies will have more room for improvement as actionable predictive biomarkers are developed.

In clinical, histological cell type is applied as a significant prognostic factor in OC, it is considered significantly related to clinical outcome of OC patients [6]. Other prognostic factors including clinical factors such as age and parity, biological factors such as multiple gene expressions and pathologic factors such as the presence of ascites and residual disease after surgery [7]. Since clinical heterogeneity and some subjective reasons, it is still hard to predict the prognosis accurately and objective now. However, as the rapid development of sequencing technologies and bioinformatic algorithms, some researchers attempt to combine multiple molecular biomarkers to established an algorithm to evaluating prognosis accurately and Clinically practically [8, 9]. In our paper, we hope to construct a risk model based on immune genes for effective prognosis prediction and throw light on targeted therapy.

Epithelial ovarian cancer (EOC) account for more than 95% of OC and was considered as an immunogenic cancer since 55% of patients was found spontaneous anti-tumor immune response [10]. The strongly link between OC and immune system can be inferred clearly. The immune system played a multifaceted role in OC and was a significant mediator of ovarian carcinogenesis [11]. Many reports proved that various immune cells and immune gene products played an integrated role in OC progression and associated with prognosis [12–14]. For better evaluating prognosis of OC patients and achieve individually management, besides histological analysis, we attempt to incorporate immune molecular features in the system of prognosis evaluation in this paper. The development of multiple immune-related prognostic markers in OC can benefit for accurate prognosis prediction, new molecular targets identification and personalized immune precision therapy until finally improve the survival rate of OC patients.

Immune-related genes (IRGs) play significant role in immune system. In this study, we aimed to build a novel immune gene signature based on IRGs for risk stratification and provide therapeutic targets in OC patients. The clinical validity and stability of the immune gene-based risk model for prognosis evaluation was validated in OC patients in the TCGA training cohort and ICGC validation cohort respectively. Our study provided an efficient and promising method for prognosis predicting and can give a valuable clue for personalized immunotherapy.

## Methods

### Data resources

The following 9 ovarian cancer (OC) expression chip datasets: GSE14407, GSE6008, GSE14001, GSE16708, GSE26712, GSE29450, GSE38666, GSE66957, and GSE105437, were downloaded from the Gene Expression Omnibus (GEO) database (www.ncbi.nlm.nih.gov/geo/) to compare the expression of 2498 immune-related genes (IRGs) in 428 OC and 77 normal ovary tissue specimens. 2498 IRGs were derived from the ImmPort database (https://www.ImmPort.org/home). The transcriptome RNA-sequencing data and corresponding clinical information of 374 and 93 OC patients were extracted from TCGA database (https://portal.gdc.cancer.gov/) and ICGC data portal (https://dcc.icgc.org/), respectively. The TCGA data and ICGC data were applied as a prognostic model training set an external validation set, respectively. Over 20,000 primary cancer samples and matched normal samples were included in the TCGA database, which held over 2.5 petabytes of genomic, epigenomic, transcriptomic, and proteomic data. The ICGC Data Portal is a collaborative effort to describe genetic anomalies in 50 different cancer types.

### Data processing and differentially expressed IRGs (DEIRGs) screening

Expression matrix data from GEO database containing 9 datasets from different labs were normalized with Limma R package. We also removed the batch effect between TCGA and ICGC datasets by SVA package in R. The differentially expressed IRGs (DEIRGs) were identified in 428 OC and 77 normal ovary tissue specimens from 9 independent GEO datasets using the limma R package, and the cutoffs were |log_2_FoldChange| (|log_2_FC|) > 1 and *p* < 0.05.

### Functional enrichment analysis and protein genes interacting of DEIRGs

The biological function of the DEIRGs was investigated using GO term enrichment analysis and KEGG pathway enrichment analysis through “clusterProfiler” R package. The protein–protein interaction (PPI) network was determined among all DEIRGs using STRING database (https://string-db.org/).

### Construction of immune gene signature related to prognosis by DEIRGs in the TCGA training cohort

First, DEIRGs with prognostic values were screened via univariate cox analysis in the TCGA training cohort. Then, to avoid overfitting, we performed the least absolute shrinkage and selection operator (LASSO) Cox regression analysis with the identified prognostic genes using R package “glmnet” to construct an immune gene signature for OC patients in the TCGA training cohort. The independent variable in the LASSO analysis was the standardized expression matrix of prognostic DEIRGs identified before, and the response variables were survival status and OS of OC patients in the TCGA training cohort. Finally, a prognostic immune gene signature for assessing survival risk of OC patients was finally constructed using the standardized expression levels of independent prognostic DEIRGs and their corresponding regression coefficients. The formula of the risk score for each patient was:

$\mathrm{The risk score}=\sum_{\mathrm{i}-\mathrm{1,2},\dots ,\mathrm{n}}\mathrm{regression coefficient }(\mathrm{genei})\times \mathrm{expression value of }(\mathrm{genei})$. OC patients were divided into low- and high-risk groups by the median risk score as the threshold.

### Evaluation of the immune gene signature in the TCGA training cohort and ICGC validation cohort

The immune gene-based risk model divide OC patients in the TCGA training cohort and the ICGC validation cohort into a high-risk group and low-risk group respectively. The Kaplan–Meier survival curves were plotted by R package “survminer” to compare the survival differences of OC patients in different risk groups. Five-year receiver operating characteristic (ROC) curves of risk score and clinical features were plotted via “survival ROC” package to describe accuracy and performance of the model in the TCGA training cohort and ICGC validation cohorts. The larger of the area under the curve (AUC) of ROC curve, the more accurate of the model. We also exhibited the relationship between survival status of OC patients and risk scores in the training cohort and validation cohort, respectively. Principal component analysis (PCA) and t-SNE analysis were performed by R package “stats” and “Rtsne” to verify the distribution of high-risk and low-risk group patients.

### Integrated analysis of the Prognostic Model and Clinical parameters of OC patients

The risk score was compared with the clinical traits to determine whether the risk score was associated with the clinical characteristics of OC patients in both TCGA training cohort and ICGC validation cohorts. Age, grade, pathological stage, and overall survival (OS) time were among the OC clinical data that were obtained from the TCGA database. The age and OS of patients were obtained from ICGC data portal. The relationship between the risk score ang these clinicopathological indexes were evaluated. To identify the independence of our risk score signature, univariate and multivariable Cox regression analyses were performed with R package “survival” in TCGA cohorts to identify independent prognostic indicators among risk score and clinical factors. Age, grade, pathological stage and Risk score were included in TCGA.

### Construction of the nomogram for OC patients

A nomogram was generated using the R package “rms” to predict the probability of 1-, 3-, 5- and 10-year OS of OC patients based on the independent prognostic DEIRGs that screened out for building the risk model.

### Correlation analysis between immune cells infiltration and immune gene signature

The immune infiltration of OC patients was derived from Tumor Immune Estimation Resource (TIMER) website (https://cistrome.shinyapps.io/timer/). The association between the abundance of 6 immune infiltrates cells (CD4+ T cells, dendritic cells, CD8+ T cells, B cells, macrophages, and neutrophils) and the immune gene-based risk model were analyzed using R.

## Results

### Screening for DEIRGs

**Fig. 1:**
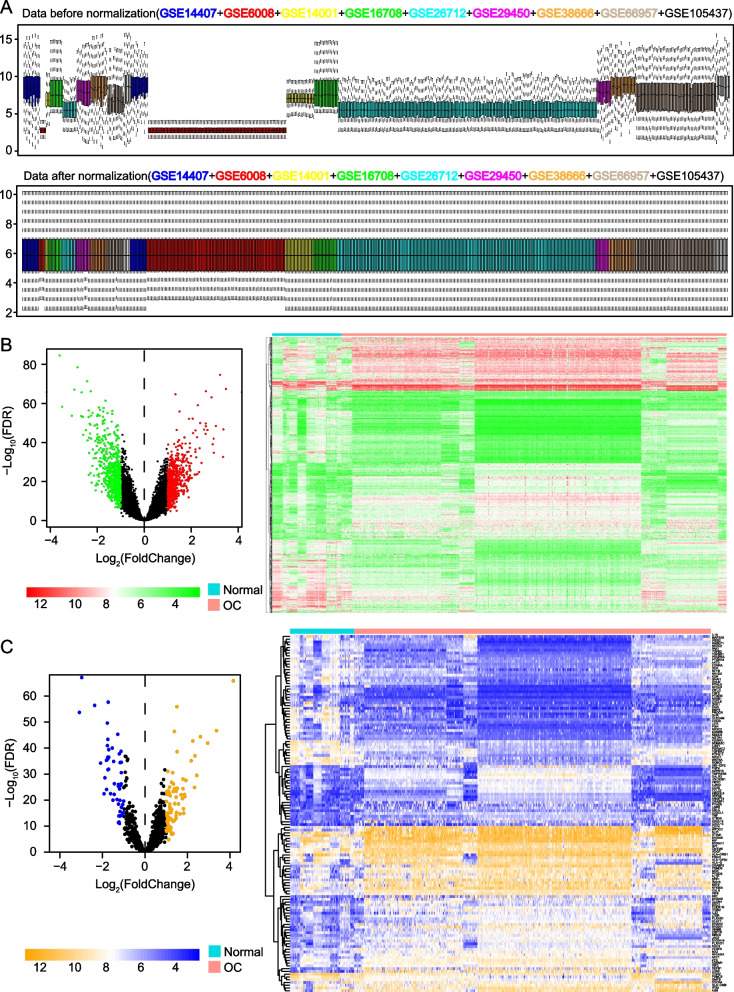
Identification of differentially expressed immune related genes (DEIRGs) between 428 OC tissues and 77 adjacent tissues from 9 independent GEO datasets. **A** Box plots of the expression profile data before and after normalization. Box plot with same color represents patients come from same GEO dataset. Left of them represents 77 control tissues and right represents 428 OC tissues. **B** Volcano plot and heatmap of differentially expressed genes. **C** Volcano plot and heatmap of DEIRGs. FDR, false discovery rate. Green and blue dots represent genes downregulated genes, red and yellow dots represent upregulated genes

After quality assessment and normalized of GEO sequencing data from 9 independent labs (Table 1, Fig. 1A), a total of 1211 gene were found to be differentially expressed genes (DEGs) in 428 OC tissues compared with 77 adjacent tissues, including 567 upregulated and 644 downregulated DEGs (Fig. 1B), we then analyzed expression of 2498 IRGs and obtained 129 differentially expressed IRGs (DEIRGs), including 79 DEIRGs and 50 DEIRGs that were upregulated and downregulated respectively (Fig. 1C).

**Table 1:** Characteristic of microarray data from GEO database that used to do difference analysis

Expression profiling array (Normal & OC)	Platforms	GEO accession	Samples
mRNA	GPL570	GSE14407	12 N; 12 OC
		GSE14001	3 N; 20 OC
		GSE29450	10 N; 10 OC
		GSE38666	12 N; 18 OC
		GSE105437	5 N; 10 OC
mRNA	GPL96	GSE6008	4 N; 99 OC
		GSE26712	10 N; 185 OC
mRNA	GPL6947	GSE16708	9 N; 17 OC
mRNA	GPL15048	GSE66957	12 N; 57 OC

### PPI network and Function Enrichment analysis of screened DEIRGs

**Fig. 2:**
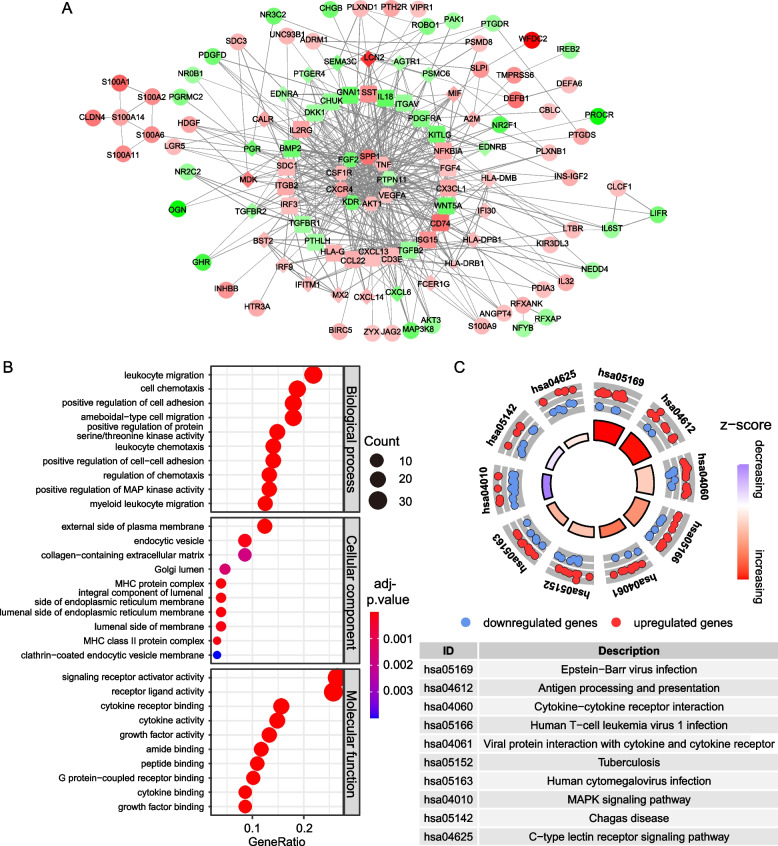
Protein-protein interaction (PPI) network, GO and KEGG functional enrichment analysis of 129 DEIRGs. **A** PPI network. Hexagonal nodes represents interactive proteins >20, square nodes represents interactive proteins >10, diamond nodes represents interactive proteins >6. Red and green nodes denote up-regulated and down-regulated DEIRGs respectively. The size of nodes is negative related to *p*-value. **B** Gene Ontology analysis (**B**) and KEGG pathway enrichment analysis (**C**) of 129 DEIRGs

A protein–protein interaction (PPI) network of DEIRGs was established and visualized in Fig. 2A. Enrichment analysis of DEIRGs showed that biological processes (BP), mainly leukocyte migration and cell chemotaxis were primarily enriched whereas the main molecular function (MF) consists of signaling receptor activator activity and receptor ligand activity. The most enriched cellular components (CC) were external side of plasma membrane (Fig. 2B). KEGG pathway indicated that the DEIRGs were mainly involved in Epstein Barr virus infection and antigen processing and presentation (Fig 2C).

### Screening of prognostic DEIRGs and Construction of a risk model based on prognostic DEIRGs in the TCGA training cohort

**Fig. 3:**
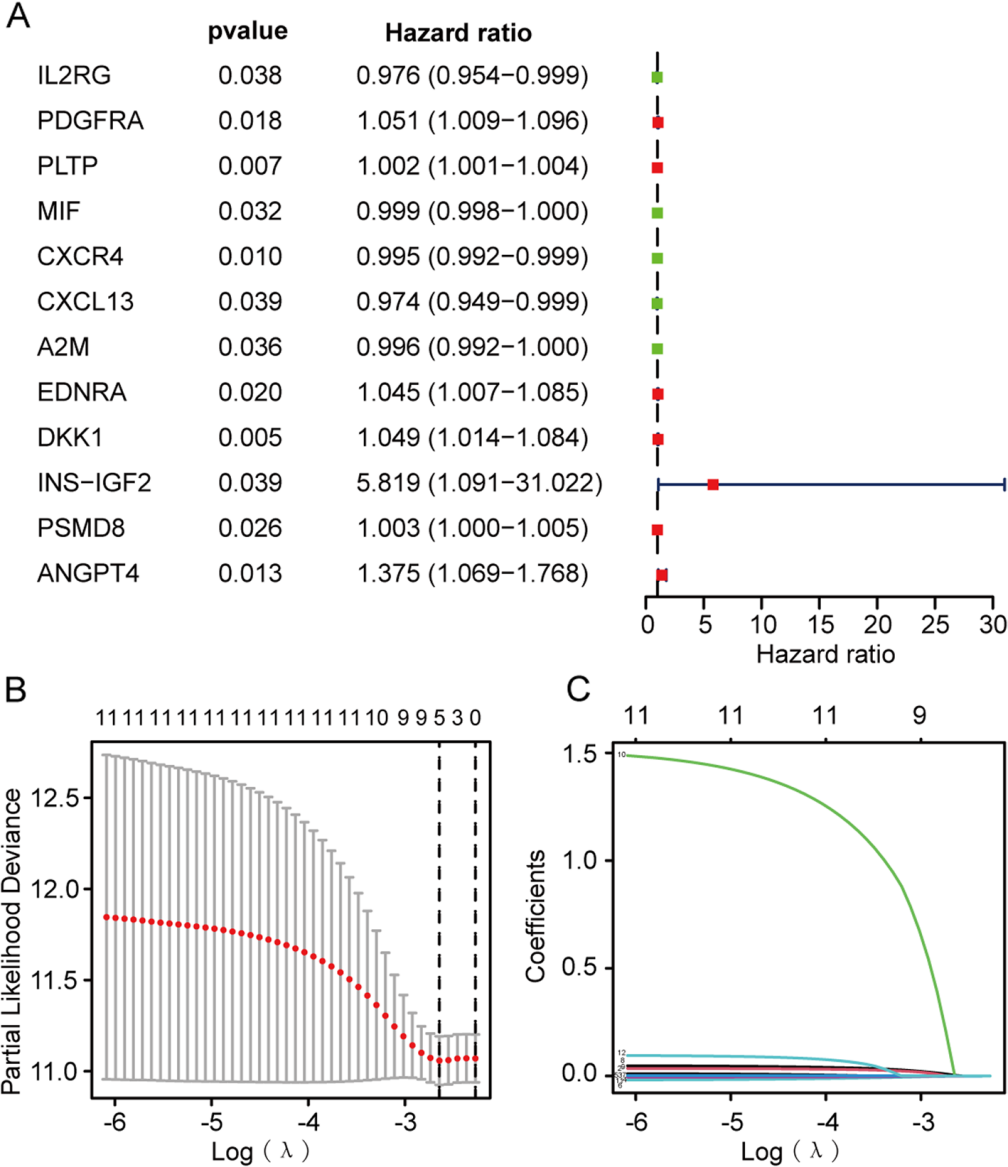
Construction of the OC-specific immune-gene based risk score system. **A** Forest plot of univariate Cox analysis showing 12 immune-related genes (IRGs) identified as prognostic factors in OC. **B** The optimal penalty parameter (λ) selection in the LASSO model. (C) LASSO coefficient profiles of the 12 survival related immune genes

We put the 129 DEIRGs obtained in the previous step into the TCGA training cohort (*n*=374) for identifying DEIRGs corelated with overall survival (OS) of OC patients. Univariate Cox regression analysis revealed 12 prognostic DEIRGs were significantly related to OS in OC patients (Fig. 3A). For avoiding overfitting, the 12 prognostic DEIRGs were included in the least absolute shrinkage and selection operator (LASSO) analysis to construct a prognostic risk model. According to the penalty parameter (Lambda) obtained in LASSO analysis, there are 5 independent prognostic DEIRGs (*ANGPT4*, *PLTP, A2M, CXCR4* and *MIF*) were used to establish a risk score model in the TCGA training set (Fig. 3B-C). The correlation coefficient of each DEIRGs make up the risk model was shown in Table 2. Risk score = (0.3697* *ANGPT4*) *+(*0.0851* *PLTP*) *+(*-0.1118* *A2M*) +(-0.0423* *CXCR4*) +( -0.0492* *MIF*).

**Table 2:** Coefficients of 5 independent key prognostic immune-related genes (IRGs) that formed the risk model

IRGs, immune-related genes	coefficients
ANGPT4	0.3697
PLTP	0.0851
A2M	-0.1118
CXCR4	-0.0423
MIF	-0.0492

### Verification of the Risk Score model in the TCGA training cohort and ICGC validation cohort

We calculated the risk score for 374 OC patients in the TCGA cohort and divided the patients into low-risk (*n*=187) and high-risk (*n*=187) groups based on the median cutoff value. The 93 OC patients in the ICGC cohort were also divided into low-risk (*n*=57) and high-risk (*n*=36) groups based on the same median cutoff value.

**Fig. 4:**
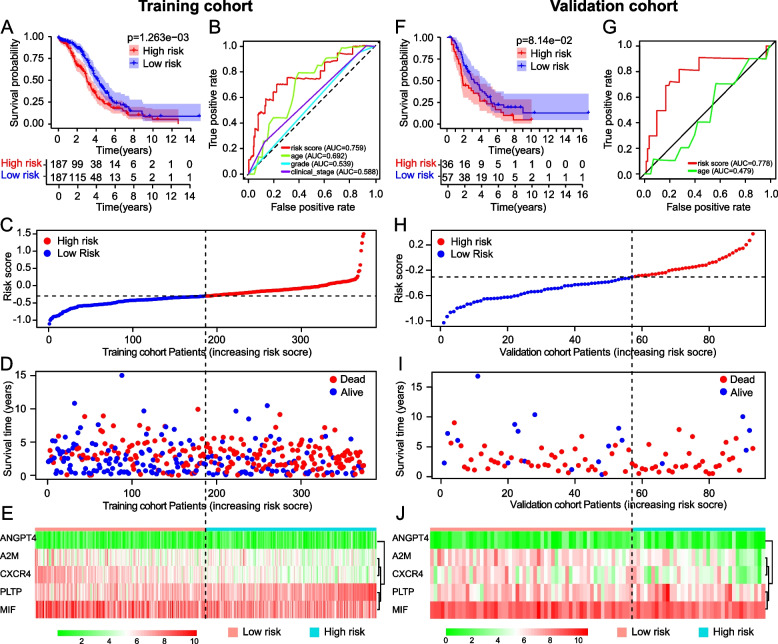
Internal and external validation of the prognostic immune gene signature in the TCGA and ICGC cohorts, respectively. Kaplan–Meier survival curves of OC patients in different risk groups in the TCGA cohort (**A**) and ICGC cohorts (**F**). AUC value and ROC curves of risk score and clinicopathologic characteristics predicting 5-year survival of OC patients in the TCGA cohort (**B**) and ICGC cohorts (**G**). The distribution of the risk score (**C**), survival time and life status (**D**), and the expression profiles of the five prognostic IRG that formed the risk model (**E**) in 374 OC patients from the TCGA cohort. **H** Distribution of 93 OC patients’ risk score in the ICGC cohort. **I** Relationship between risk score, survival time and life status in the ICGC cohort. **J** The expression of 5 risk genes in the ICGC cohort

Figure 4A exhibited that in 374 OC patients from TCGA cohort, the OS of low-risk patients was markedly higher compared to that of high-risk patients. The 5-year ROC curve of Risk score and other clinical parameters were plotted to assess the reliability of the risk model, and the areas under the curve (AUCs) of risk score is 0.759, higher than any other clinical parameters (Fig. 4B). The risk score distribution of OC patients in the TCGA training cohort is shown in Fig. 4C, as the risk score increased, increase in number of deaths occurred (Fig. 4D). The expression patterns of 5 prognostic DEIRGs that composed the risk model in the TCGA training cohort was visualized (Fig. 4E).

The stability of the immune gene-based risk model was also examined in the ICGC-validation cohort. Consistent with the TCGA training cohort, OC patients in the low-risk group showed a substantially higher survival rate (Fig. 4F). In the results of the 5-year ROC curve of the ICGC validation cohort, the AUC value of the risk score was 0.778 and the significance for evaluating the prognosis far exceeded other clinical indicators (Fig. 4G). The risk score performed well not only in the training cohort but also in the validation cohort (TCGA-AUC = 0.759, ICGC-AUC = 0.778). Risk score distribution and corresponding survival status of OC patients in the ICGC validation cohort were presented in Fig. 4H-I. The expression profile of 5 risk genes in ICGC cohort was also exhibited in Fig. 4J.

All above results suggested that the model had good accuracy and general applicability.

### Evaluation of clinical practicality of the immune gene-based risk model

**Fig. 5:**
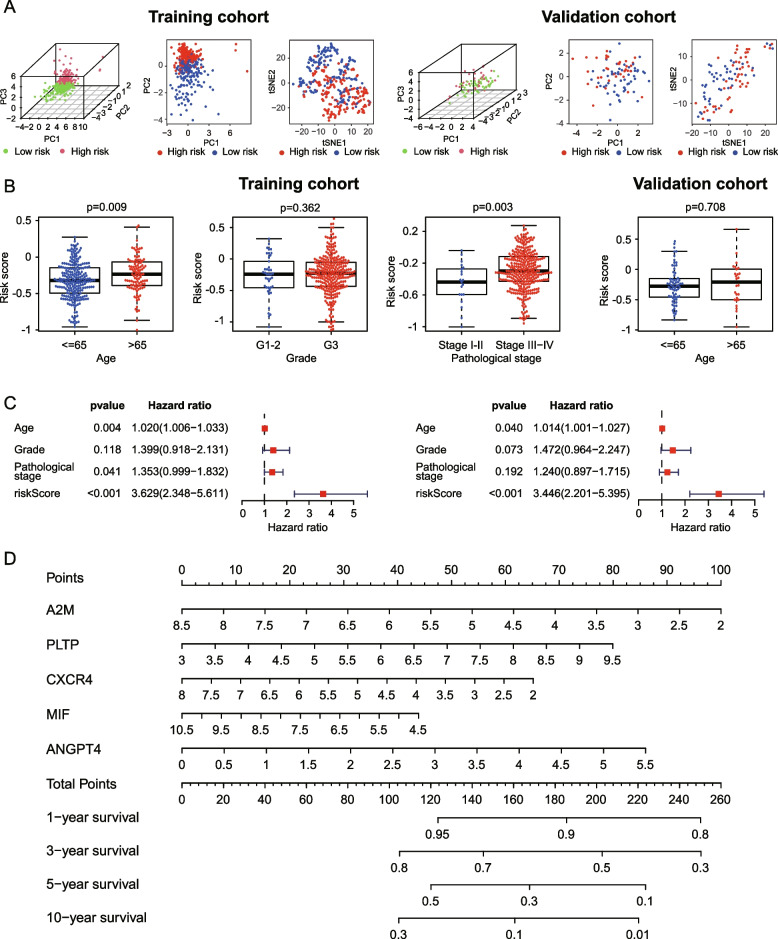
The clinicopathological significance of the prognostic risk model, and a nomogram for predicting OS of OC patients. **A** Principal component analysis (PCA) and t-distributed stochastic neighbor embedding (t-SNE) analysis showing patients in high-risk and low-risk groups in TCGA dataset (left panel) and ICGC dataset (right panel) were distributed in 2 discrete directions. **B** Age and pathological stage were significantly associated with the risk score in the TCGA training cohort. **C** The univariate (left panel) and multivariate (right panel) Cox regression analyses of clinical parameters and immune risk signature of OC patients in the TCGA cohort showing risk score can be used as independent prognostic judgment factors. **D** The prognostic nomogram for predicting the survival probability of OC patients based on the TCGA training cohort

Complete clinicopathological data were extracted and integrated with risk score of OC patients in TCGA cohort and ICGC cohort respectively, and evaluated whether the risk score could independent irrespective of other clinical features to be a prognostic factor. We used principal component analysis (PCA) and t-distributed stochastic neighbor embedding (t-SNE) analysis for data dimensionality reduction and found that patients in high-risk and low-risk groups in TCGA dataset (left panel) and ICGC dataset (right panel) were distributed in 2 discrete directions (Fig. 5A). The corresponding scatter diagrams determined by the Wilcoxon Rank Sum Test showed that age and pathological stage of patients in the TCGA training cohort were significantly related to the risk score. No significant differences were observed between various grade groups in the TCGA dataset or different age groups in ICGC dataset (Fig. 5B). All clinicopathological parameters and risk score were subjected to a univariate independent prognostic analysis, the result reflected age and risk score were significantly associated with the OS of the patients in the TCGA training cohort (Fig. 5C, left panel). Multivariate analysis including age, grade, pathological stage, and riskScore. The multivariate independent prognostic analysis results exhibited that the risk score was the only one independent prognostic judgment factor (Fig. 5C, right panel). To construct and visualize a survival prediction method for OC patients, a prognostic nomogram including 5 risk genes which made up the risk model was developed (Fig. 5D).

### Correlation between immune gene-based risk model and immune cell infiltration

**Fig. 6:**
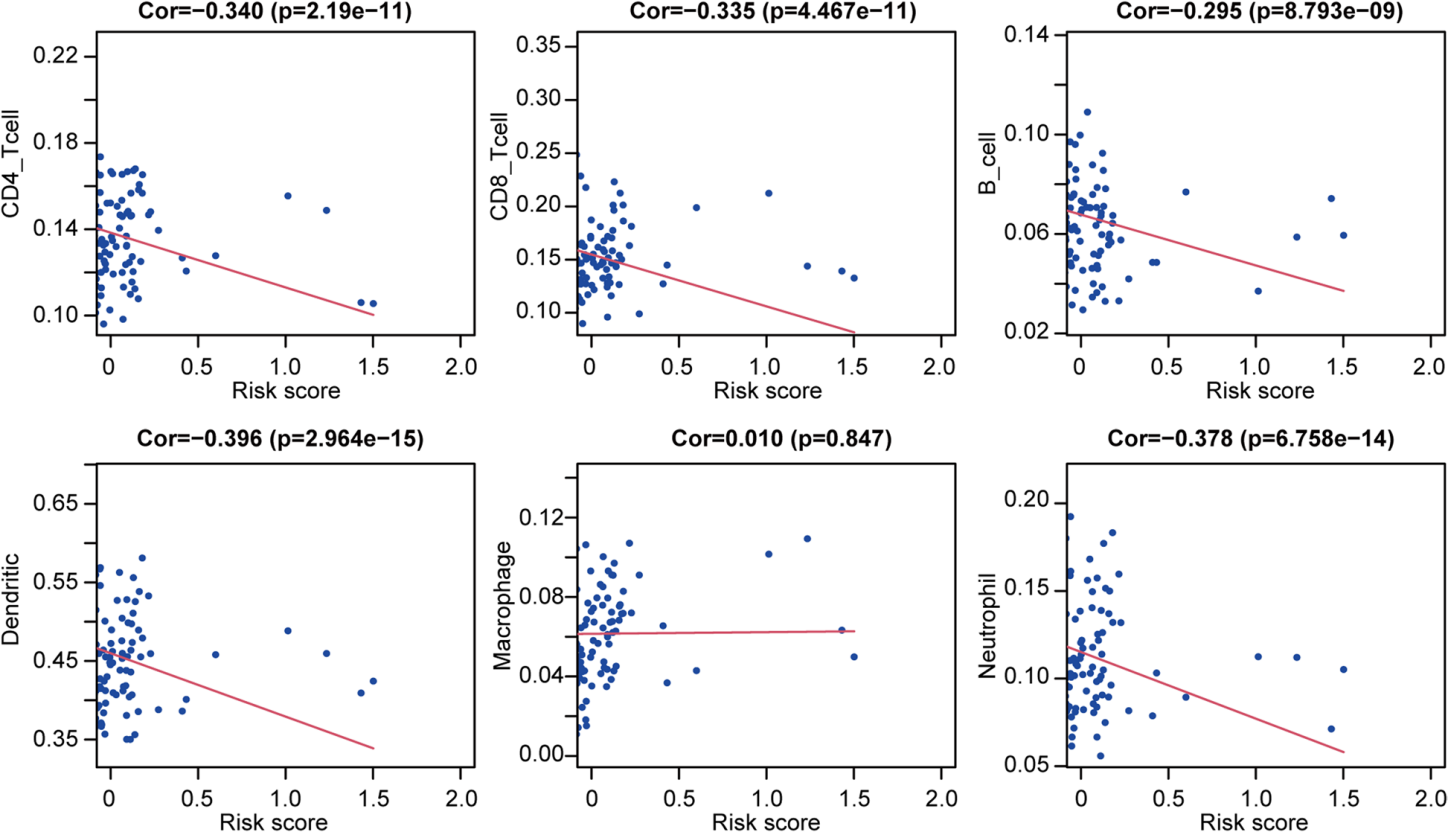
Correlation of the immune gene based prognostic model with infiltration abundances of 6 types of immune cells

To evaluate whether our risk score model could reflect the tumor immune microenvironment in OC patients, we analyzed the relationship between risk score and immune cell infiltration in the TCGA training dataset. As show in Fig. 6, CD4+ T cells, CD8+ T cells, B cells, dendritic cells, and neutrophils were negative correlated with risk score. It indicated that the level of immune cell infiltration was downregulated in high risk OC patients.

## Discussion

Ovarian cancer represented 2.5% of all female malignancies, but lead to 5% mortality among all cancer deaths. The high mortality of OC was mainly due to 80% of patients were diagnosed at an advanced stage with extensive peritoneal cavity metastases [15, 16]. For these patients diagnosed at an advanced stage, surgery and chemotherapy are still the standard of care [17]. Since the responses of different patients to treatment is diversity, this reminder us to searching for highly reliable prognostic biomarkers. Efficiency prognostic biomarkers are conducive to distinguish patients at different levels of risk, convenient for treatment choice, and facilitate patient counseling [18].

At present, it had become a hotspot to establish gene signatures based on specific characteristics for prognosis predicting in cancer research [19, 20]. Immunoediting is a process present in OC, it comprised of cancer cell elimination, equilibrium and escape from immune surveillance, and was a significant element of the immune system [21]. The immune system plays a significant and complicated role in OC, it has been proved [22]. Klemi et al confirmed that T cells in colorectal cancer specimens can predicted the outcome more accurately than standard prognostic factors [23]. Other studies also showed similar results [24]. These studies proved the significance of the immune response in prognosis. Although there are some researchers want to explore the relationship between OC and immune response from different perspectives, such as using ceRNA that affecting immune infiltration [25], or using macrophage-related gene [26] or immune-related gene pairs [27] to construct a risk model, our study using immune-related genes to expound the relationship between OC and immune response is more immediately and comprehensive. Our risk model was composed of only 5 risk genes, and verified in 2 independent cohort, the novel risk prediction model based on immune-related genes for OC patients was verified the accuracy and clinical validity from several aspects. Our study is a novel research that construct an immune genes signature for prognosis evaluating, and can provide clues to targeted therapy with immune related genes.

With the development of precision genomic medicine, researchers are committed to identify specific and accurate prognostic factors from massive medical data sets with clinical outcomes [28]. A multigene-based model for prognosis predicting was obviously more precise and robust compared with using a single gene [29, 30]. To evaluate prognosis by expression of 5 immune genes in OC patients is convenient, efficient, accurate and cost-effective. We constructed an immune genes signature for OC patients for the first time. There are some studies studied the relationship between 5 risk genes (*ANGPT4* [31], *PLTP* [32]*, A2M* [33]*, CXCR4* [34] and *MIF* [35]) that composed the immune genes signature and OC. However, our study constructed a risk model using the 5 risk genes firstly, can with the model, we can predict prognosis for OC patients more accurate than only one biomarker. According to our study, risk score may offer correct risk classification as a standalone prognosis factor, according to prognosis analysis on the risk model. Therefore, our nomograms built on DEIRG-based prognostic markers can help OC patients better quantify their risk. Meanwhile, ANGPT4 and PLTP were high risk DEIRGs while A2M, CXCR4 and MIF were low risk DEIRGs, maybe they are potential therapeutic targets, of course more study should be done in future.

Of course, although our risk model based on immune genes can predict the prognosis of OC patients rather good, there are many other factors associated with the prognosis of OC patients, including metabolism, autophagy and so on. Therefore, further prospective studies should be implemented in multicenter clinical trials. All in all, for the first time, this study established and validated a novel immune gene related prognostic model using strict standards. It may contribute to the development of individualized treatments and improve OC patients’ OS.

## Conclusions

Our research successfully constructed and validated a risk score model composed of 5 immune-related mRNAs which had superior predictive capacities to predicted prognosis of OC patients, it can be used in clinical decision making and guide the personalized immunotherapy.

### Supplementary Information

**Additional file 1: Supplementary Table 1.** Demographic and clinical details of OC patients in TCGA cohort.

**Additional file 2: Supplementary Table 2.** Demographic and clinical details of OC patients in ICGC cohort.

## Data Availability

The raw data supporting the conclusions of this manuscript will be made available by the authors, without undue reservation, to any qualified researcher.

## Abbreviations

AUC: Area under the curve
DEIRGs: Differentially expressed Immune ‑ related genes
GO: Gene Ontology
ICGC: International Cancer Genome Consortium
IRGs: Immune ‑ related genes
KEGG: Kyoto Encyclopedia of Genes and Genomes
KM plotter: Kaplan-Meier plotter
LASSO: Least absolute shrinkage and selection operator
Log_2_FC: Log_2_Fold Change/Logarithm of Fold Change
OC: Ovarian cancer
OS: Overall survival
PPI: Protein-protein interaction
TCGA: The Cancer Genome Atlas
