# Supplementary material for: Novel immune-related gene signature for risk stratification and prognosis prediction in ovarian cancer
Source: J Ovarian Res. 2023 Oct 19;16:205. doi: 10.1186/s13048-023-01289-w (PMC10585734; doi:10.1186/s13048-023-01289-w)
Supplement: Supplementary file 1 — Additional file 1: Supplementary Table 1. Demographic and clinical details of OC patients in TCGA cohort. [file 13048_2023_1289_MOESM1_ESM.docx]

| Characteristics | Number | Percentage (%) |
| --- | --- | --- |
| Age (years) |  |  |
| <=65 | 257 | 68.7% |
| >65 | 117 | 31.3% |
| Vital status |  |  |
| Alive | 170 | 45.5% |
| Dead | 204 | 54.5% |
| Histologic grade |  |  |
| G1-2 | 43 | 11.8% |
| G3 | 321 | 88.2% |
| Pathological Stage |  |  |
| I-II | 23 | 6.2% |
| III-IV | 348 | 93.8% |

Supplementary Table 1: Demographic and clinical details of OC patients in TCGA cohort.
