# Supplementary material for: Novel immune-related gene signature for risk stratification and prognosis prediction in ovarian cancer
Source: J Ovarian Res. 2023 Oct 19;16:205. doi: 10.1186/s13048-023-01289-w (PMC10585734; doi:10.1186/s13048-023-01289-w)
Supplement: Supplementary file 2 — Additional file 2: Supplementary Table 2. Demographic and clinical details of OC patients in ICGC cohort. [file 13048_2023_1289_MOESM2_ESM.docx]

| Characteristics | Number | Percentage (%) |
| --- | --- | --- |
| Age (years) |  |  |
| <=65 | 67 | 72.0% |
| >65 | 26 | 28.0% |
| Vital status |  |  |
| Alive | 19 | 79.6% |
| Dead | 74 | 20.4% |

Supplementary Table 2: Demographic and clinical details of OC patients in ICGC cohort.
